# Atherosclerosis, Diabetes Mellitus, and Cancer: Common Epidemiology, Shared Mechanisms, and Future Management

**DOI:** 10.3390/ijms241411786

**Published:** 2023-07-22

**Authors:** Vasiliki Katsi, Ilias Papakonstantinou, Konstantinos Tsioufis

**Affiliations:** 1 Department of Cardiology, Hippokration Hospital, 11527 Athens, Greece; ktsioufis@gmail.com; 2 4th Department of Internal Medicine, Evangelismos Hospital, 10676 Athens, Greece; iliaspapacon@yahoo.gr; 3 School of Medicine, National and Kapodistrian University of Athens, 11527 Athens, Greece

**Keywords:** lipoprotein metabolism, cancer metabolism, endothelial dysfunction, metabolic syndrome, oxidized lipids, oncogenic signals, autophagy, oxidative stress, adiposity, glycation, atherosclerosis

## Abstract

The involvement of cardiovascular disease in cancer onset and development represents a contemporary interest in basic science. It has been recognized, from the most recent research, that metabolic syndrome-related conditions, ranging from atherosclerosis to diabetes, elicit many pathways regulating lipid metabolism and lipid signaling that are also linked to the same framework of multiple potential mechanisms for inducing cancer. Otherwise, dyslipidemia and endothelial cell dysfunction in atherosclerosis may present common or even interdependent changes, similar to oncogenic molecules elevated in many forms of cancer. However, whether endothelial cell dysfunction in atherosclerotic disease provides signals that promote the pre-clinical onset and proliferation of malignant cells is an issue that requires further understanding, even though more questions are presented with every answer. Here, we highlight the molecular mechanisms that point to a causal link between lipid metabolism and glucose homeostasis in metabolic syndrome-related atherosclerotic disease with the development of cancer. The knowledge of these breakthrough mechanisms may pave the way for the application of new therapeutic targets and for implementing interventions in clinical practice.

## 1. Introduction

Atherosclerosis and cancer, among the most common human diseases, are determined by the result of underlying genetic predisposition and lifetime exposure to multiple factors that threaten a healthy life [1,2]. Atherosclerosis, a chronic inflammatory disease of the arterial wall, leads to the formation of atherosclerotic plaques and cardiovascular disease [3]. Atherosclerotic cardiovascular disease (ASCVD) is recognized to be substantially driven by important aspects of metabolic syndrome. Metabolic syndrome is characterized by a set of cardiovascular risk factors that include abdominal obesity, insulin resistance, impaired glucose metabolism, hypertension, and dyslipidemia [4]. Above all, the increased proportion of atherogenic small, dense low-density lipoproteins (LDLs) in metabolic syndrome, even though LDL-cholesterol levels may be optimal, reflects their direct involvement in the chronic inflammatory processes of atherosclerosis [5].

Clinical and epidemiological studies have linked defects in lipid metabolism to cancer and describe the association of several types of cancers with risk factors for cardiovascular disease [6,7,8]. Moreover, metabolic syndrome, as a cluster of risk factors for ASCVD and type 2 diabetes, has been substantially associated with the onset of malignancies [9]. For example, patients with metabolic syndrome have an increased risk of atherosclerosis-related cancer compared to metabolically healthy individuals and show an increased incidence and aggressiveness of tumor formation [10,11,12].

While our understanding of multiple factors continues to evolve, the interaction between metabolic syndrome, ASCVD, and cancer appears to be challenging to study, because ASCVD and cancer share multiple risk factors, and questions are raised about the actual contribution of each to cancer risk [13]. It is important to note that ASCVD risk factors like smoking, a diet high in saturated fat, a higher intake of sugar and high-glycemic-index foods, a sedentary lifestyle, and a lack of exercise are modifiable and subject to prevention [14]. How and to what extent these risk factors contribute to cancer is likely unknown. For instance, artificial sweeteners (especially aspartame and acesulfame-K), which are used in beverages, are associated with an increased cancer risk [15]. Similarly, human studies link the composition of dietary fat to the pathogenesis of cancer rather than the total fat content in isocaloric diets [16]. It is also likely that genetic predisposition, under the influence of environmental and atherogenic factors, may be implicated in both cardiovascular diseases and cancer and may be the initiating event in tumorigenesis–malignant transformation [17]. Moreover, while it has been shown that LDL retention is crucial for the initiation of atherosclerosis, its contribution to the malignancy of cancer is not known [18].

To uncover the processes that link ACVD and cancer, “chronic inflammation”, as the first step, is typically considered the leading mechanism [19,20]. Chronic inflammation, a container concept of different inflammatory networks, combines cellular and humoral pathways, which are also intertwined with additional molecular mechanisms and metabolic parameters [3,21,22].

First of all, the milestone event in atherosclerosis development is endothelial cell (EC) activation in susceptible vascular areas, regarded as “athero-prone”, at arterial branch points and curvatures, where blood flow turns from laminar and unidirectional to oscillatory, thus resulting in minimal but continuous shear stress on ECs [23]. At these sites of highly dynamic and inflamed endothelial microenvironments induced by hemodynamic stress, LDLs are deposited and are oxidized to form highly atherogenic oxidized LDL (oxLDL) [24]. LDL oxidation is mediated by reactive oxygen species (ROS) produced by oxidative phosphorylation (OXPHOS) in the mitochondria of damaged ECs [3,25]. Then, macrophages are chemoattracted and take up oxLDL with the help of scavenger receptors, forming foam cells. Foam cells secrete cytokines, which trigger a series of inflammatory reactions and progression to atheromatous plaques [26].

In addition to oxidation and chronic inflammation, angiogenesis has been demonstrated to be an important driving force of atherosclerosis and thus ASCVD. The predominant angiogenic mechanism in atherosclerotic lesions is sprouting angiogenesis from pre-existing vasa vasorum [25]. Angiogenic factors specific to atherosclerotic angiogenesis may include the vascular endothelial-specific growth factors VEGF-A, angiopoietins, and hypoxia-inducible factor-1α (HIF-1a) [25]. Furthermore, signaling pathways are activated by oxLDL and ROS in the subendothelial space to drive the expression of proinflammatory cytokines as mediators in atherosclerosis progression. Among them, toll-like receptor (TLR), NLRP3 inflammasome, Notch, and Wnt signaling, when dysregulated, play an important role [21]. Most of these signaling pathways interfere with transcription factors like nuclear transcription factor κB (NFκB), which mediates the production of interleukins related to inflammation and atherogenesis [27,28].

While the contribution of inflammation is central in cardiovascular diseases, the link between inflammation and cancer is less understood than the connection between inflammation and atherosclerosis. Inflammation has been recognized for its roles in cancer initiation, invasion, and progression [29,30,31]. The gain of oncogenes and loss of tumor suppressors are key characteristics of cancer cells. One of the most well-known oncogenic regulators, the tumor suppressor *p53* can also regulate cellular metabolism [32]. In addition, deregulated NF-κB activity causes inflammation-related diseases, as well as cancers [33]. Moreover, the same molecular families of inflammation, specifically those targeting the pathways regulating lipid metabolism and atherogenesis, are elevated in many forms of cancer, and they provide growth signals that promote the proliferation of malignant cells [21,34].

The production of ROS, besides being an important aspect of ASCVD, also represents one of the hallmarks of tumors, as it occurs in response to the hypoxic (low oxygen level) tumor microenvironment (TME) [35]. This causes excessive abnormal angiogenesis, which plays a pivotal role in tumor progression, a process driven by the tissue hypoxia-triggered overproduction of VEGFs [36]. To increase oxygen delivery to the hypoxic environment, transcription factors are activated; among them, HIF-1a, which is also implicated in atherosclerotic angiogenesis, promotes alternative metabolic pathways that regulate the adaptation, survival, and aggressiveness of tumor cells [37]. Increased cancer risk is also associated with dysfunctional visceral fat, which plays a key role in the initiation and maintenance of chronic inflammation, fibrosis (extracellular matrix hypertrophy), and impaired angiogenesis, as well as consequent unresolved hypoxia [38]. In adipose tissue, lipids and accumulating lipid droplets (LDs) are common phenotypic features of dyslipidemia in metabolic syndrome [39]. Of interest, white adipose tissue (WAT) inflammation is associated with metabolic syndrome and pro-neoplastic genes [40]. Moreover, cancer cells exhibit the presence of abundant LDs, which suggests that the storage of lipids may be a common feature of malignancies [41].

In this context, at present, it is not clear whether atherosclerosis triggers cancer or whether there is a true cause–effect association. Moreover, questions remain on how the molecular guardians of cell metabolic homeostasis divert cells into malignant transformation. From this perspective, we review the molecular signaling and mechanisms that point to a causal link between the development of cancer and metabolic abnormalities associated with lipid metabolism in ASCVD. This article is not intended to provide detailed information about all the important key areas of cancer metabolism because of the countless molecular particles involved. Beyond this, the paucity of clinical data linking atherosclerosis to cancer and, most importantly, regarding successful metabolic therapies for patients with cancer invigorate foundational science efforts. Therefore, we highlight recent key developments in the field. For the purpose of this review, we first explain important aspects of endothelial dysfunction and metabolic alterations of the endothelium, which have been implicated in the pathophysiology of cancer and atherosclerosis. Then, we describe prominent features regarding the metabolic aberrations of cancer cells. From various sources in the literature, we catalog the oncogenic pathways implicated in atherosclerosis and cancer, and we discuss whether angiogenic and lipogenic factors act as drivers of tumor initiation and how they may be involved in the metabolic rewiring of cancer cells [21,42]. In this regard, we further discuss the role of adiposity and adipose tissue LDs in malignant progression. Toward the end of this review, we assess the role of hyperglycemia in metabolic syndrome and diabetes mellitus in malignant transformation, with an emphasis on advanced glycation end products (AGEs). Finally, we discuss the potential use of metabolism-modulating drugs as a strategy to combat cancer.

Considering the common, shared mechanisms in metabolic syndrome-associated ASCVD and cancer, we hope to expand the fundamental knowledge on this topic and present a clinical point of view, accompanied by molecular evidence, with the aim to support clinicians in optimizing human health and implementing interventions into clinical applications.

## 2. Input from Epidemiology

Atherosclerosis eventually leads to cardiovascular disease (ASCVD), a leading cause of global morbidity and mortality because of potentially hazardous complications, i.e., acute myocardial infarction (MI), stroke, and ischemic cardiomyopathy [43,44]. Estimated ASCVD-attributed deaths are near the top, accounting for one-third of all deaths [45]. Silent coronary atherosclerosis without an established disease is common in the general population, as shown in a large, random sample of a middle-aged population from Sweden without previous myocardial infarction or coronary intervention, where coronary computed tomography angiography (CCTA) showed that 42.1% of the population had plaques in their coronary arteries. Significant stenosis (≥50%) was less common (in 5.2%), and more severe forms of coronary atherosclerosis, like three-vessel disease, were rarely found (in only 1.9%) [46]. The prevalence of CCTA-detected coronary atherosclerosis in this study closely reflects the incidence of ASCVD in many other Western countries [46,47,48]. Cancer is also a very common disease. According to the WHO’s (World Health Organization) data, the incidence of cancer has been rising in recent years, representing the second leading cause of death globally, with major mortality and economic impacts [49]. The situation is so alarming that every fourth person has a lifetime risk of cancer [50].

### 2.1. Epidemiological Studies

Metabolic syndrome, affecting 13% of the general population, is reported to increase the risk of developing cancers like colorectal, endometrial, and postmenopausal breast cancer [51]. However, questions remain as to whether multiplicative and additive interactions exist between atherosclerosis-related metabolic health status on cancer risk [51,52]. For example, exposure to cigarette smoke per se represents a major risk factor for atherosclerosis by activating several mechanisms, including thrombosis, insulin resistance and dyslipidemia, vascular inflammation, abnormal vascular growth and angiogenesis, and loss of endothelial homeostatic and regenerative functions [53]. Even though there is an apparent direct association between smoking and cancer, if there are additional factors in cancer outcomes through different pathways, neither the causality nor the strength of the associations can be estimated [54]. Connections between cancer and ASCVD, metabolic complications, and genetic influences have become more established through genome-wide association studies called Mendelian Randomization (MR) studies [55]. The MR approach to overcoming the limitations of traditional observational study designs utilizes the random allocation of genotypes at conception, which makes genotypes independent of potential confounders while also avoiding reverse causation [54,56,57].

### 2.2. Dyslipidemia and Cancer Risk

Epidemiological studies have demonstrated that LDL and oxLDL are associated with breast cancer, colorectal cancer, pancreatic cancer, and other malignancies, suggesting that LDL and oxLDL play important roles during the occurrence and development of various cancers, for example, breast cancer, ovarian cancer, and colorectal cancer [58]. An association between dyslipidemia and increased cancer risk was observed in some studies [59]. While meta-analyses found that high dietary intake of cholesterol increased the risk of esophageal cancer, pancreatic cancer, and ovarian cancer, epidemiological data on cancer risk and lipid levels are contradictory for some types of cancer [60].

### 2.3. Diabetes and Cancer Risk

Many observations suggest that obesity and diabetes are associated with an increased risk of developing several types of cancers, including liver, pancreatic, endometrial, colorectal, and postmenopausal breast cancer. Recent observational evidence shows a robust association between type 2 diabetes and colorectal, hepatocellular, gallbladder, breast, endometrial, and pancreatic cancers [61]. Potential causal associations were identified for genetically predicted type 2 diabetes and fasting insulin concentrations and the risk of endometrial, pancreas, kidney, breast, lung, and cervical cancers [61]. A causal effect of higher fasting insulin, but not glucose traits or type 2 diabetes, on increased colorectal cancer risk has been demonstrated [62]. This finding is supported by the inclusion of MR studies in diabetes where genetically predicted type 2 diabetes and/or fasting insulin levels, rather than genetically predicted fasting glucose levels, were associated with the risk of cancer in the uterus, kidney, pancreas, and lung [63]. An MR study investigating the causal associations of type 2 diabetes with the risk of overall cancer and 22 specific sites found causal effects on several cancers, like pancreatic, kidney, uterine, and cervical cancer, and lower odds of esophageal cancer and melanoma, but no association with 16 other site-specific cancers or overall cancer [63]. However, an MR analysis used to clarify the relationship between diabetes and cancer in a Japanese cohort of 32,949 individuals, including 3541 incident cancer cases, found no convincing evidence to support associations between diabetes and overall and site-specific cancer risks, suggesting that there is little evidence to support the genetic role of type 2 diabetes in cancer development in this population [64].

### 2.4. Obesity and Cancer Risk

Metabolic syndrome, as a cluster of conditions including obesity and metabolic aberrations, has been shown to be associated with an increased risk of some obesity-related cancers, like pancreatic, postmenopausal breast, liver, colorectal, endometrial and renal cell cancer [65]. Epidemiological evidence shows that about 4–8% of all cancers are attributed to obesity and that obesity is a risk factor for several major cancers, including postmenopausal breast, colorectal, endometrial, kidney, esophageal, pancreatic, liver, and gallbladder cancer [66,67]. Excess body fat results in an approximately 17% increased risk of cancer-specific mortality [68]. There are two types of obesity, one with metabolic aberrations or metabolic unhealthy obesity (MUO) and another with normal metabolic status (MHO) [65]. The combination of obesity and metabolic aberrations in metabolic syndrome has been consistently associated with an increased risk of several established obesity-related cancers [65]. Although evidence remains limited for separate cancers, MHO does not appear to be a benign condition and is associated with an increased risk of several cancers, approximately 30% higher for any obesity-related cancer compared with metabolically healthy, normal-weight individuals [65].

## 3. Insight into Endothelial Dysfunction in Atherosclerosis

Endothelial cells (ECs) line the lumen surface of blood vessels and are metabolically active to maintain vascular homeostasis [69]. Furthermore, the endothelium plays a critical role in the regulation of whole-body metabolism, and the modulation of EC metabolism can markedly affect systemic glucose and lipid homeostasis [70,71]. ECs can rapidly switch between specific metabolic pathways in response to changes in the extracellular environment [71]. From a metabolic viewpoint, ECs are glycolytic; in fact, ECs retrieve more than 85% of their energetic needs from aerobic glycolysis, while only a small amount of pyruvate generated during this process is used to fuel OXPHOS [72]. The choice of relying on glycolysis allows ECs to deliver more oxygen to the surrounding tissues as well as to minimize the amount of harmful ROS compared to OXPHOS, and furthermore, because glycolysis is faster in terms of ATP (adenosine triphosphate) synthesis compared to OXPHOS, it plays a crucial role in angiogenesis when tip and non-tip cells differentiate within the new sprout [69]. In addition, the enzyme 6-phosphofructo-2-kinase/fructose-2,6 biphosphatase (PFKFB3), a molecular regulator of glycolysis, has been found to be directly involved in the metabolic switch of ECs by inducing the migratory phenotype of tip cells [73].

At the beginning of the atherosclerotic process, continuous shear stress on ECs from oscillatory blood flow at sites regarded as “athero-prone”, like arterial branch points and curvatures, disturbs the endothelial function [74]. This endothelial dysfunction alters EC metabolism via HIF-1a, which induces their proliferation and inflammation by activating glycolytic enzymes, conditions supporting the initiation of atherosclerosis [75]. Endothelial dysfunction is characterized by an increase in the secretion of proinflammatory cytokines and by an increase in ROS levels [76]. ROS are produced in cells as a result of normal physiological processes in oxidative stress, which in turn may lead to DNA damage and alterations in tumor suppressor genes, and eventually, this may contribute to the initiation, development, and progression of cancer [77]. The evidence that links aberrant EC metabolism to vascular dysfunction and metabolic diseases is strengthened by a recent study showing that the deficiency of endothelial transcription factor EB (TFEB) leads to impaired glucose tolerance via reduced Akt signaling and reduced insulin receptor substrate 1 and 2 expression. This study uncovered a novel role of TFEB in EC metabolism and identified TFEB as a potential therapeutic target for treating various vascular and metabolic diseases [78]. TFEB regulates homeostasis in the cardiovascular system and has beneficial effects on ASCVD [79] through its proangiogenic, anti-atherosclerotic, and anti-inflammatory effects on ECs [80]. Aberrant, i.e., excessive or insufficient, neovascularization could lead to the onset of pathological conditions, including diabetes [81], cardiovascular disease [25], and cancer [82,83]. PFKFB3, as one of the EC glycolytic pathway’s key regulators, by also controlling the balance of tip versus stalk cells in sprouting angiogenesis, may represent a potentially druggable target [36].

## 4. A Look at Modulations in Cancer Metabolism

To better understand cancer, we should briefly review the literature on the metabolic deviations that occur in cancer cells and the surrounding tumor microenvironment/TME overall.

### 4.1. Metabolic Reprogramming

Normal differentiated cells depend on mitochondrial OXPHOS for the energy required for cellular processes under a precisely controlled system to prevent abnormal growth, whereas cancer cells mainly depend on aerobic glycolysis [72]. The dysregulation of cellular metabolism has now reemerged as an important driver of cancer [84]. In cancer cells, metabolism must also be reprogrammed to support the energy requirements of the biosynthetic processes and to enable them to proliferate, overcome apoptosis, and promote invasion and metastasis [22]. The reprogramming of these metabolic pathways in cancer cells is characterized by metabolic switching from mitochondrial OXPHOS to high rates of glycolysis and lactate production, which promotes tumor growth and the increased metabolism of glutamine (glutaminolysis) [85]. High rates of glycolysis are traditionally associated with hypoxia, a driver of HIF-1a, which in turn increases glycolysis and tumor development under anoxic conditions [86]. In tumors, glycolysis can also be upregulated by HIF-1a even when oxygen is abundantly present in the surrounding tissues consisting of the TME; this has been suggested to impair the progression and recurrence of tumors and the formation of metastasis [87]. Both the increased OXPHOS pathway and the development of the chronic hypoxic condition inevitably contribute to the increased production of ROS in cancer. The reason why cancer cells choose the less efficient glycolysis is that glycolysis is much faster than OXPHOS, allowing cells to occupy advantageous positions when they are competing for shared energy resources to support cellular anabolic reactions [88].

ROS are important signaling molecules in cancer, promoting the evolution of early cancer cells into more malignant cancerous cells as they progress into the later stage, while oncogenes drive the transformation of cancer cells from normal cells [85]. The major species of ROS generated in cancer are superoxide (O_2_^−^), hydrogen peroxide (H_2_O_2_), the hydroxyl radical (^−^OH), and lipid hydroperoxide (LOOH) [89,90]. However, because the rate of ROS production in cancer is much higher in comparison to normal cells, to limit the damaging effects of ROS, cancer cells utilize several mechanisms to use localized H_2_O_2_ for pro-tumorigenic signaling while simultaneously maintaining a high antioxidant capacity to detoxify damaging ROS molecules such as O_2_^−^, ^−^OH, and LOOH [91].

### 4.2. Autophagy

Another strategy for the survival of cancer cells occurs by activating autophagy. In this condition, cancer cells recycle intracellular components in conditions of metabolic stress and, by undergoing epithelial–mesenchymal transition (EMT), can gain resistance to cell death when spreading outside the tumor mass [92,93]. Autophagy is an intracellular self-defense mechanism of normal cells too, where organelles and proteins are degraded into autophagy bubbles, thereby preventing the toxic accumulation of damaged or unnecessary components but also recycling these components to sustain metabolic homeostasis [94,95]. Recent research has demonstrated that TFEB, which acts on endothelial cells, participates in the regulation of autophagic flow, and by increasing autophagy, TFEB may be essential for cancer cell proliferation and spread [80].

## 5. Basic Oncogenic Pathways Dysregulated in Atherosclerosis

Metabolic disorders are involved in the aberrant dysregulation of oncogenic signaling. Several studies have found that numerous oncogenic signaling pathways affect cholesterol production, implying that they play a role in tumorigenesis. When chronically activated, these pathways can drive malignant transformation and may represent a link between metabolic syndrome, ASCVD, and cancer. Moreover, cancer cells co-opt signaling pathways and transcriptional networks to increase metabolism, sustain proliferation, and promote cancer cell growth, survival, motility, and drug resistance [96,97]. Further, we described increased activities of oncogenic signals observed in patients with metabolic disorders and atherosclerosis [30].

### 5.1. Τhe PI3K–Akt–mTOR Signaling Pathway

The phosphoinositide 3-kinase–protein kinase B (PI3K-Akt) pathway is the most commonly activated pathway, which, under physiological conditions, is activated in response to insulin, growth factors, insulin-like growth factor (IGF), VEGF, and cytokines to regulate key metabolic processes [98,99,100]. PI3K-Akt pathway activation mediates the high demand for cellular nutrients in cancer cells and reprograms cellular metabolism by providing nutrients, promoting glycolysis, protein and lipid synthesis, and OXPHOS, and regulating autophagy [101,102]. mTOR is a serine/threonine protein kinase and is composed of two distinct multiprotein subunits, mTORC1, activated by PI3K-Akt, and mTORC2, activated by growth factors [102,103]. Both complexes contribute to cancer metabolic reprogramming. mTORC1 induces cell growth and facilitates glycolysis, OXPHOS, glutaminolysis, and protein translation through 4E-binding protein (4E-BP) and the translation initiation factor eIF4E and lipid synthesis [88]. Interestingly, active mTORC1 phosphorylates TFEB, thus causing its inactivation and inhibiting its nuclear translocation [104]. However, in nutrient-deprived conditions, TFEB becomes active and free from phosphorylation, translocates to the nucleus, and increases the expression of genes involved in autophagy–lysosomal biogenesis; furthermore, it induces the expression of peroxisome proliferator-activated receptor gamma co-activator 1-alpha (PGC1a), a known central regulator of mitochondrial biogenesis and fatty acid oxidation [104,105]. In nutrient-rich conditions, mTORC1 is activated on the lysosomal surface, inhibits autophagy, and supports anabolism, a finding that has led to the resurgence of research into lysosomal biology [95,104,106]. Constitutively enhanced TFEB may promote tumorigenesis [104]. mTORC2 plays critical roles in context- or subset-specific metabolic reprogramming for cell growth by inducing Akt phosphorylation and activation (PI3K-Akt signaling activation) and, therefore, promoting tumor survival [107]. Akt activation upregulates sterol regulatory element-binding protein 1 (SREBP1) gene expression, and it also inhibits the ubiquitin–proteasome pathway degradation of SREBP1 through protein arginine methyltransferase 5 (PRMT5), inducing SREBP1 hyperactivity, which results in de novo lipogenesis and tumor growth [108]. Diabetes mellitus is involved in the dysregulation of the PI3K–Akt–mTOR oncogenic pathway, which is responsible for most metabolic and mitogenic effects of insulin [109].

### 5.2. AMPK

Adenosine monophosphate-activated protein kinase (AMPK), a serine/threonine kinase, serves as a central guardian of energy homeostasis and metabolism by orchestrating diverse cellular processes, such as lipogenesis, glycolysis, cell cycle progression, and mitochondrial dynamics [110,111]. AMPK favors cellular catabolism by promoting mitochondrial fitness, driving mitochondrial biogenesis and fission, as well as the clearance of damaged mitochondria via mitophagy and autophagy [112]. Under energy-deprived conditions, AMPK is activated, triggering autophagy [95]. AMPK is activated by energy stress because of glucose/glutamine deprivation and because of an increase in the cellular AMP–ATP ratio (low ratio of adenylate kinase AMP/ADP-to-ATP) due to decreased ATP production and, consequently, elevated levels of intracellular AMP [113,114]. The activation of AMPK is also manifested by the upstream kinases LKB1 (liver kinase B1) and CaMKK2 (calcium calmodulin kinase 2), which phosphorylate AMPK [113].

LKB1 activates AMPK in case of energy deprivation [115,116]. LKB1, which was genetically identified to be a tumor suppressor [117,118], is a broad regulator of cellular metabolism, impacting lipid, cholesterol, and glucose metabolism in the liver, muscle, and adipose tissue. Specifically, LKB1-AMPK inhibits fatty acid and cholesterol synthesis by phosphorylating the metabolic enzymes HMG-CoA reductase (HMGCR) and acetyl-CoA carboxylase 1 (ACC1) [119]. CaMKK2 is identified as a critical rheostat for the regulation of hepatic glucose and lipid metabolism [102,113,120]. AMPK is dysregulated in diabetes, obesity, cardiometabolic disease, and cancer.

In atherosclerotic CVD, emerging evidence supports a pleiotropic and overall protective effect for AMPK by reducing ROS formation and inflammation and by inhibiting immune cell adhesion, foam cell formation, and vascular smooth muscle cell (VSMC) proliferation, all central events in the development of atherosclerosis [121]. AMPK also plays a critical role in maintaining glucose homeostasis and improves insulin sensitivity [122].

In cancer, AMPK demonstrates an enigmatic role [117,123]. AMPK senses low cellular energy and can either repress or promote tumor growth depending on the context [124]. AMPK activation in nutrient-depleted conditions maintains both autophagy and lysosomal function in cancer to promote survival [125]. AMPK activation inhibits tumorigenesis by regulating signaling pathways such as PI3K, mTOR, and *p53*, which are involved in cellular proliferation, cell cycle progression, and cellular survival [118]. The loss of function of LKB1 due to mutations and the loss of AMPK activity are found in various cancers [125]. From recent observations in an animal model, the AMPK/mTORC1 and Nicotinamide Adenine Dinucleotide Phosphate (NADPH) oxidase enzyme 4 (NOX4) signaling axis was highlighted as a critical molecular mechanism linking hyperglycemia to colorectal cancer [126]. Also, acting through SREBP1, the LKB1-AMPK pathway inhibits lipogenesis, thereby inhibiting cell growth and tumorigenesis [119].

### 5.3. Toll-Like Receptors (TLRs)—TLR Signaling Pathway

The toll-like receptors (TLRs) are a class of transmembrane proteins with a cytoplasmic region homologous to the IL-1 receptor and an extracellular domain; they act as pattern recognition proteins and play an integral role in the modulation of systemic inflammatory responses through their modulatory effects on various immune cells and the release of various inflammatory mediators [21,127]. TLR stimulation triggers myeloid differentiation factor 88 (MyD88) to interact with interleukin-1 receptor-associated kinase 4 (IRAK4), which transmits signals into NF-kB to activate the expression of inflammatory cytokines via the MyD88-dependent pathway [21,128].

#### 5.3.1. Role of TLRs in Atherosclerosis

In atherosclerosis, dysregulation of TLRs is a key mechanism for inflammation, contributing to the development of cardiovascular diseases [129]. Indeed, several TLRs, particularly TLR2, TLR4, and TLR9, are found in the endothelial cell membrane and are involved in endothelial dysfunction as well as in the development and progression of atherosclerosis [130]. Potential major antigens and neoepitopes released when oxLDL is formed in the vessel wall in response to tissue stress or damage are highly immunogenic and recognized by TLRs, which are then upregulated and transmit signals to NF-kB to activate the expression of inflammatory cytokines via the MyD88-dependent pathway [131].

#### 5.3.2. Role of TLRs in Cancer

Several studies have demonstrated that TLRs are upregulated in different neoplasias, such as breast, lung, pancreatic, and colon cancer, where they are associated with a favorable or poor prognosis [132]. Prolonged and uncontrolled activation of TLR by chronic inflammatory stimulation may in turn alter the proliferative patterns of the cells, which ultimately leads to the development of cancer [133]. As such, TLRs may serve as a hub for crosstalk during cancer progression. Upon activation, TLRs, through different pathways, lead to the transcription factor NF-kB, which supports the inflammatory microenvironment [134]. Moreover, high-mobility group box 1 (HMGB1), a key ligand for TLRs, interacts with TLR2 and TLR4 to provoke inflammatory responses via the NF-kB pathway [135,136]. Furthermore, HMGB1 release activates TLR-4 and increases IL-1 production through Nod-like receptor protein 3 inflammasome activation [137].

### 5.4. NLRP3 Inflammasome Pathway

Inflammasomes are multimeric protein complexes that are part of the innate immune response and are assembled in response to molecular danger signals, with the Nod-like receptor protein 3 inflammasome, or the NLRP3 inflammasome, being the most widely studied [138]. Endogenous factors and mechanisms identified to promote NLRP3 assembly and activation include ROS, hypoxia, metabolites, oxLDL, and signals from TLRs [138,139]. Transient receptor potential melastatin 2 (TRPM2), an oxidative-stress-sensitive calcium channel, has also been implicated in the regulation of the NLRP3 inflammasome [140].

#### 5.4.1. Role of NLRP3 Inflammasome in Atherosclerosis

NLRP3 is involved in the pathogenesis of atherosclerotic cardiovascular disease [141]. In atherosclerosis, endogenous danger signals by cholesterol crystals and by oxLDL are released and trigger NLRP3 formation, a process induced by TLRs through MyD88 [142]. The binding of oxLDL to cluster of differentiation 36 (CD36), the formation of the CD36-TLR4-TLR6 complex, and the internalization of oxLDL results in lysosomal damage [139]. In the next step, NF-κB is activated and upregulates the transcription of NLRP3 and pro-IL-1β, which promotes the assembly and activation of NLRP3 [143]. Activated NLRP3 oligomerization recruits caspase, ultimately leading to the proteolytic cleavage of pro-IL-1β and pro-IL-18 into their active forms [144]. IL-1β, as a proatherogenic cytokine, is involved in the initiation, formation, and growth of atheromatous plaques, suggesting that it is a key element in atherosclerotic pathogenesis [145]. NLRP3 dysfunction can contribute to many diseases, including diabetes [138], and plays a pivotal role in activation and endothelial dysfunction, especially aggravating oxidative stress and ROS, which in turn act as an intermediate trigger to activate NLRP3 [146]. The role of the NLRP3 inflammasome in the pathogenesis of atherosclerosis, however, warrants further investigations.

#### 5.4.2. Role of NLRP3 Inflammasome in Cancer

The roles of NLRP3 in cancer are complicated and rather contradictory. Multiple studies have shown the involvement of NLRP3 in tumorigenesis, whereas others have indicated a protective role in certain cancers [147]. It is possible that autophagy dysfunction can lead to diseases with hyper-inflammation and the excessive activation of NLRP3, which in turn may contribute to the development of cancer [148]. Furthermore, a study in mice deficient in NLRP3 components showed exacerbated liver colorectal cancer metastatic growth, linking innate immune system inflammasome activation to effective NK-cell-mediated tumor attack for the suppression of colorectal cancer growth in the liver [149]. Moreover, TRPM2, a regulator of NLRP3, is overexpressed in many malignancies [150].

### 5.5. Notch—The Notch Pathways

Notch is a cellular signaling pathway that mediates the significant role of Notch in vascular shaping and maturation during angiogenesis [151]. This is because Notch has the ability to determine the tip cell phenotype, which guides the nascent sprout to develop by responding to VEGF [36,151]. Accumulating evidence has demonstrated that Notch protects against the dysfunction of the endothelium caused by inflammatory cytokines, inhibiting ECs differentiation into tip cells, therefore maintaining the connection between ECs and endothelial integrity [152]. The Notch pathway consists of at least five transmembrane ligands, two Jagged ligands (Jagged 1, 2) with different numbers of epidermal growth factor (EGF)-like repeats, with the latter mediating receptor–ligand binding, and three Delta-like ligands (DLLs)/Delta-like ligands 1, 3, and 4 that bind to four different transmembrane receptors (Notch 1, 2, 3, and 4), which initiate Notch signaling when the Notch receptor interacts with its ligand located on an adjacent cell [153,154]. The Notch pathway modulates mitochondrial function to promote cell survival by activating mTORC2/Akt signaling with PTEN-induced kinase 1 (PINK1) [155].

#### 5.5.1. Notch Regulates Atherosclerosis

The activity of Notch signaling in various cell types is influenced by inflammatory lipids, suggesting a link between the metabolic status and Notch signaling activity [156]. In atherosclerosis, M1 inflammatory macrophages sustain mechanisms that favor atherosclerosis progression, whereas M2 macrophages drive mechanisms that can suppress plaque formation and progression and even support plaque regression [157]. By controlling the differentiation of macrophages into the proinflammatory M1 or anti-inflammatory (or less-inflammatory) M2 subtype, the DLL4-Notch1 axis promotes the polarization of M1 macrophages and blocks M2 polarization in the development of atherosclerosis [155]. A recent study demonstrated that the inhibition of DLL4–Notch signaling by anti-DLL4 improves elevated glucose, stimulates insulin secretion, and improves islet function and insulin production by multiple complementary mechanisms, suggesting DLL4-Notch as a therapeutic target for improving islet function and glucose regulation in diabetes [158].

#### 5.5.2. The Implication of Notch Signaling in Cancer

Dysregulation of Notch signaling is increasingly associated with different types of cancer, and proteins in the Notch signaling pathway can act as oncogenes or tumor suppressors, depending on the cellular context and aberrant tumor type [153,159]. Moreover, cell-to-cell signaling has a specific implication in cancer development via angiogenesis, primarily through DLL4 in the Notch signaling pathway [160,161]. Notch-targeted therapy has been studied for decades but has failed to meet expectations. The reasons for these shortcomings might be the cytotoxicity induced by pan-Notch inhibitors and the upregulation of bypass pathways [152].

### 5.6. Wnt—The Wnt Pathway

Wnt signaling also regulates cell survival, growth, and motility and can initiate the transcriptional co-activator β-catenin, which participates in cell adhesion [162]. Wnt activation of β-catenin leads to target gene upregulation via the family of T-cell factor/lymphoid enhancer factor (TCF/LEF) transcription factors [163]. Wnt/β-catenin can be activated by transforming growth factor-β (TGF-β) signaling and interact in the fibrosis process [164].

#### 5.6.1. Role of Wnt Signaling in Atherosclerosis

Aberrant Wnt signaling plays a significant role in the pathogenesis of atherosclerosis [165]. Low-density lipoprotein receptor-related proteins 5 and 6 (LRP5 and LRP6) act as co-receptors of Wnt ligands and are indispensable for Wnt signal transduction, preventing the cytoplasmic degradation of β-catenin [166]. Patients carrying a mutation in the Wnt co-receptor LRP6, a member of the LDLR gene family, exhibit elevated levels of LDL cholesterol, triglycerides, and fasting glucose, which cooperatively constitute risk factors for metabolic syndrome and atherosclerosis [167,168]. Beyond regulating cell proliferation and differentiation, the Wnt pathway also controls lipid homeostasis and storage [165]. LRP5 is also found to prevent atherosclerosis [169].

#### 5.6.2. The Wnt Pathway in Cancer

The aberrant activation of Wnt/β-catenin signaling is a critical factor for primary transformation and tumor growth to metastasis and cross-communication among cancer cells [162]. Wnt signaling promotes EMT via crosstalk with TGF-β signaling cascades, while the TGF-β network induces immune evasion [170]. Previous studies also showed that β-catenin interacts with HIF-1a in multiple physiological and pathological processes [171]. Further, Wnt/β-catenin signaling activates PI3K/Akt, which stimulates HIF-1a-induced metabolic reprogramming under non-hypoxic conditions and promotes cancer cell survival [172]. In accordance with previous studies demonstrating the role of hyperglycemia in carcinogenesis, a recent study has shown that hyperglycemia can induce Wnt/b-catenin signaling, promoting cancer cell survival and the progression of hyperglycemia-related cancer [173]. Taken together, the Wnt signaling pathway is an emerging biological link between ASCVD, diabetes, and cancer and may be a novel therapeutic target in the future.

## 6. Angiogenic Factors in Atherosclerotic Disease and Cancer

Angiogenesis is a normal physiological mechanism defined as the formation of new blood vessels and capillaries from already existing ones; this condition is called “sprouting” [174]. The angiogenic front of the sprouting vessel is characterized by two EC phenotypes, stalk and tip ECs, but the key event in sprouting angiogenesis is the selection of motile leading-edge tip cells [25,36]. Poorly perfused ECs exposed to high VEGF concentrations extend numerous filopodia and become tip cells, initiating sprouting angiogenesis. The degradation of the basal membrane and the detachment of mural cells then result in stalk cells [69]. While angiogenesis is considered an important mechanism for the development of atherosclerotic disease [25], it also represents one key event in several types of cancer [175]. This is because cancer is associated with a highly hypoxic state, which promotes hypoxic-tumor-derived transcription factors [176,177,178]. The most widely known angiogenic factors are the family of vascular endothelial growth factors (VEGF-A, VEGF-B), VEGF receptors (VEGFRs), and angiopoietins [179]. Other factors that promote angiogenesis and tumor expansion include HIF-1a (hypoxia-inducible factor-1a) and nuclear factor erythroid 2-related factor 2 (NRF2) [180]. The imbalance between angiogenic and antiangiogenic factors promotes an “angiogenic switch”, triggering blood vessel formation and angiogenesis [36].

### 6.1. Endothelial Growth Factor (VEGF) and VEGF Receptors (VEGFRs)

Endothelial growth factor (VEGF) promotes the proliferation, migration, and spread of endothelial cells and is an undisputed crucial player in EC activation for both physiological and pathological angiogenesis [36,181,182]. VEGF triggers cell responses by recruiting the tyrosine kinase receptors VEGFR1/Flt-1, VEGFR2/KDR/Flk1, and VEGFR3/Flt-4, and among them, VEGFR2 is the most potent mediator of changes that occur during VEGF-induced tip cell selection [69].

#### 6.1.1. VEGF-Mediated Signaling

The activation of the VEGF receptor by its ligand promotes signaling pathways important to endothelial cell proliferation, including phosphoinositide-3-kinase (PI3K), protein kinase B (PKB/Akt), and mitogen-activated protein kinase (MAPK)/extracellular signal-regulated kinase (ERK) pathway (p38-MAPK/ERK1/2), facilitating the migration of inflammatory cells and the release of inflammatory cytokines and proteolytic enzymes into the extracellular matrix (ECM) [183,184]. Moreover, under hypoxic stress, DLL4-NOTCH reacts and represses VEGFA-VEGFR2 signaling, inhibiting their differentiation into tip cells [69,152].

Important cell receptors for VEGF/VEGFR signaling in angiogenesis include TFEB and Neuropilin 1 (NRP1). TFEB relies on the catalytic activity of VEGF receptor 2 (VEGFR2) to regulate EC proliferation [185,186]. Vascular NRP1, as a receptor, participates in distinct types of signaling pathways that control cell migration in angiogenesis and has been shown to synergize with VEGF-A as a co-receptor for VEGFR2 [183,187,188].

#### 6.1.2. VEGF/VEGFR in Atherosclerosis

VEGF is part of the classical angiogenic factors that mediate atherosclerotic angiogenesis, in addition to its role in neovascularization, plaque growth/progression, and instability [25]. VEGF has a multifactorial influence on energy homeostasis, the lipidemic profile, insulin resistance, glucose sensitivity, and cardiac function [189]. The effects of VEGFs on the development of atherosclerosis are complex and diverse, but VEGF-A prevents the repair of endothelial damage, contributing to atherogenesis and promoting monocyte adhesion, transendothelial migration, and activation [188]. VEGF-A, as part of the VEGF-A/VEGFR-1-NRP1 signaling pathway, regulates chylomicrons entering the chylous duct; thus, dysregulation could cause the inhibition of chylomicron absorption [190]. Also, VEGF-A decreases the activity of plasma lipoprotein lipase (LPL), resulting in the accumulation of triglycerides in chylomicrons and very low-density lipoproteins, which results in atherosclerosis promotion [191]. On the other hand, VEGF-B is known for its lipid-lowering effect. Acting via VEGFR-1/AMPK and NRP-1, VEGF-B regulates the transcription of vascular fatty acid transporters, thus controlling the uptake of fatty acids from circulating lipids by ECs and their further transcytosis, followed by lipid utilization in mitochondria [192]. In addition, VEGF-B signaling impairs the recycling of low-density lipoprotein receptors (LDLRs) to the plasma membrane, leading to reduced cholesterol uptake and membrane cholesterol loading, which also leads to a decrease in glucose transporter 1 (GLUT1)-dependent endothelial glucose uptake [193].

#### 6.1.3. VEGF/VEGFR in Cancer

The expression of VEGF and VEGFRs is upregulated in solid tumors, and this significantly contributes to the formation of tumor blood vessels, leading to cancer development and dissemination [194]. Following the discovery that many tumors secrete VEGF, it is not unexpected that the VEGF pathway has been considered one of the most attractive targets for the development of antiangiogenic drugs [195]. The clinical benefit of drugs targeting the VEGF/VEGFR pathway, neutralizing monoclonal antibodies, has been modest for most tumor types; nevertheless, combinations of VEGF/VEGFR pathway inhibitors with immune checkpoint blockers have attained new interest [36,196].

### 6.2. Angiopoietins

Angiopoietins consist of a small group of secreted glycoproteins that are implicated not only in the normal process of angiogenesis, regulating vascular permeability and the growth, modification, and recovery of the blood vessels, but also in pathological vascular remodeling during inflammation, tumor angiogenesis, and metastasis [36,197]. In humans, angiopoietins constitute a small group of three secreted glycoproteins named Angiopoietin-1 (Ang-1), Angiopoietin-2 (Ang-2), and Angiopoietin-4 (Ang-4) (the mouse ortholog is named Ang-3), which are ligands of the Tie-2 tyrosine kinase receptor [198]. Ang-1 is regarded as a strong Tie-2 agonist and promotes vessel maturation and survival through Tie-2 receptor phosphorylation and via the PI3K-Akt-mediated signaling pathway [83]. Ang-2 has been regarded as an antagonist of Tie-2 that competitively restrains Ang-1 binding. However, more recent studies imply that Ang-2 has a dual impact on angiogenesis since it acts either as a weak Tie-2 agonist or as a Tie-2 antagonist [36,199].

#### 6.2.1. Angiopoietins in Atherosclerosis

Angiopoietin-1 may play a proatherogenic role, while angiopoietin-2, which acts as an antagonist of Angiopoietin-1, may inhibit atherosclerosis by limiting LDL oxidation [200]. In line with this remark, in hypercholesterolemic LDLR−/−Apolipoprotein B (ApoB)100/100 mice, anti-angiopoietin-2-blocking antibodies exerted an anti-atherogenic effect [201]. Moreover, in mice, Ang-2 showed increased expression in lesions with intraplaque hemorrhage compared to regions of the lesions without [202]. From other experiments in mice, when Ang-4 protein was injected twice a week into atherosclerotic ApoE−/−mice, Ang-4 reduced atherosclerotic plaque size and vascular inflammation and inhibited atherogenesis [203]. Another study showed that genetic ablation of Ang-4 in adipose tissue results in enhanced plasma lipoprotein lipase (LPL) activity, the rapid clearance of circulating triacylglycerols, increased lipolysis and fatty acid oxidation, and decreased synthesis, suggesting that a lack of Ang-4 in adipose tissue enhances the clearance of proatherogenic lipoproteins, attenuates inflammation, and reduces atherosclerosis. [204].

#### 6.2.2. Angiopoietins in Cancer

It has been estimated that predominantly Ang-2 is extensively expressed in tumor endothelial cells and, in association with VEGF and other proangiogenic factors, triggers tumor angiogenesis [36]. This is accompanied by the proteolytic degradation of the basement membrane by matrix metalloproteinases (MMPs), which results in the loosening of endothelial cell–cell junctions [36]. Nowadays, it is well established that the serum levels of Ang-2 are significantly associated with the onset and progression of non-small-cell lung cancer (NSCLC) [197]. Ang-2 transcription augments the migration, invasion, and EMT of lung cancer cells [205]. While the role of Ang-4 (which acts as an agonist of the Tie-2 receptor) in tumor angiogenesis and invasion seems unclear, Ang-4 was recently shown to be associated with cancer progression by promoting glucose metabolism in colorectal cancer [206]. 

### 6.3. Nuclear Factor Erythroid 2-Related Factor 2 (NRF2)

NRF2 belongs to the group of redox-sensitive transcription factors expressed in several tissues and, by promoting ROS detoxification, improves oxidative stress and maintains redox homeostasis [207]. Kelch-like ECH-associated protein 1 (KEAP1) is an inhibitor of NRF2 [208], while heme oxygenase-1 (HO-1) is a target of NRF2, which induces its expression [209].

#### 6.3.1. NRF2 Signaling in Atherosclerosis

Based on the mentioned data, the NRF2 signaling pathway is currently considered an important defense mechanism against ASCVD; however, the mechanisms underlying the preventive effects of NRF2 are barely known. The nuclear factor erythroid 2-related factor 2/heme oxygenase-1 (NRF2/HO-1) pathway confers antioxidant effects and plays a crucial protective role in cellular responses to oxidative stress, which is a risk factor for ASCVD, due to the degradation of pro-oxidant heme and the generation of antioxidants biliverdin and bilirubin [209].

#### 6.3.2. NRF2 Signaling in Cancer

NRF2 has paradoxical roles in cancer biology, either acting as a tumor suppressor or exerting oncogenic effects [210]. There are also conflicting data as to whether NRF2 promotes or inhibits tumor initiation because no studies have demonstrated that NRF2-activating mutations alone are sufficient to initiate cancer [210]. It is known that cancer cells, to limit the damaging effects of ROS, utilize the transcription factor NRF2 to upregulate antioxidant proteins [91]. This may be mediated through the activation of the pentose phosphate pathway (PPP), a major glucose catabolic pathway, which redirects glucose and glutamine into anabolic processes, especially under the sustained activation of oncogenic signaling [211]. During the early phases of tumorigenesis, ROS appear to be mutagenic, and therefore, they support cell transformation into cancer cells [84]. Evidence indicates that ROS increase upon transformation, but their levels are kept in check by antioxidant systems, as orchestrated by NRF2 [212]. It is possible that limiting ROS is necessary for the initiation of cell transformation, whereas sustaining ROS levels promotes metastasis [91]. Likely, during tumor progression, a different type of ROS is being affected, and high levels of toxic ROS (that is, O_2_^−^, ^−^OH, LOOH) may be a barrier to tumor initiation; thus, initiation requires the elevated expression of both NRF2 activation and TP53-induced glycolysis and apoptosis regulator (TIGAR) to support toxic ROS scavenging [91]. Robust evidence for the importance of ROS in cancer comes from human cancer genetic analysis and studies showing that loss-of-function mutations in cytoplasmic KEAP1 result in the activation of NRF2 in the context of other cancer-promoting mutations [213]. Hence, the NRF2 regulation of the antioxidant response by eliminating ROS and maintaining a normal redox state can have a detrimental impact on cancer treatment [214]. NRF2/KEAP1 may also protect against aberrant inflammation by regulating the uncontrolled activation of the NF-κB pathway, which can result in inflammatory cell damage and lead to malignant cell transformation [215]. NRF2 upregulates HO-1, which, besides removing toxic heme, produces biliverdin, iron ions, and carbon monoxide and thus exerts beneficial effects by protecting against oxidative injury, the regulation of apoptosis, and the modulation of inflammation, as well as the contribution to angiogenesis [216]. However, the role of HO-1 in tumorigenesis has not been systematically addressed, although emerging data show the multiple roles of HO-1 in tumorigenesis, from pathogenesis to the progression to malignancy, metastasis, and even resistance to therapy [217].

### 6.4. Hypoxia-Inducible Factor-1a/HIF-1a

Hypoxia-induced factor 1-alpha (HIF-1a), a heterodimeric protein part of the basic helix-loop-helix family, is regarded as the core molecule of an oxygen-sensing mechanism in the body, which is hypoxia [218,219].

#### 6.4.1. HIF-1a in Atherosclerosis

HIF-1a in atherosclerosis exerts both detrimental and beneficial actions, depending on the cell type expressing HIF-1a [220]. OxLDLs can trigger HIF-1a activation through TLRs in macrophages, with consequences for interleukin-1β (IL-1β) production and the metabolic rewiring of macrophages with the induction of glycolysis (rather than OXPHOS) [220]. Overall, HIF-1a plays a key role in the critical steps of atherosclerosis development, acting on endothelial cells, vascular smooth muscle cells, and foam cell formation, and through the upregulation of VEGF, ROS, and the NF-kB pathways in endothelial cells, HIF-1a is able to cause endothelial cell dysfunction, angiogenesis, and inflammation [221].

#### 6.4.2. HIF-1a in Cancer

HIF-1a, activated by hypoxia, is highly expressed in the TME and represents the main trigger for the growth of new blood vessels in malignant tumors [222]. HIF-1a induces the upregulation of angiogenic factors, VEGF/VEGFR, and angiopoietin with the Tie2 receptor at the transcriptional level, thereby promoting the formation of new blood vessels in cancer, leading to tumor growth, progression, and metastasis [180]. HIF-1a is also directly linked to leptin, which shows strong proangiogenic properties [223,224]. HIF-1a levels are regulated by multiple signaling pathways that play an important role in human cancer, among them the PI3K-Akt-mTOR signaling pathway [101]. PI3K/Akt signaling upregulates HIF-1a transcription and translation by PI3K/Akt signal, regardless of oxygen levels [225,226]. Both PI3K/Akt activity and HIF-1a expression are influenced by ROS. Collectively, these results suggest that ROS-induced high HIF-1a expression is, to a certain extent, mediated via PI3K/Akt activation [227]. HIF-1a is a key regulator of cancer metabolism. Deregulation of HIF-1a coupled with the abnormal expression of metabolic enzymes (pyruvate dehydrogenase complex) during cancer development might play a role in inducing the deviation of tumor cells from the default OXPHOS program to enter into a permanent aerobic glycolytic metabolic pathway as an adaptation to low oxygen tension, as the main metabolic pathway for generating ATP, even in the presence of oxygen [228].

## 7. The Role of Lipogenic Factors in Atherosclerosis and Cancer

Lipoproteins and lipid factors, important in the pathophysiology of atherosclerosis, that have been shown to play a pro-tumorigenic role in several cancers include oxidized LDL (oxLDL), Lectin-Like Oxidized Low-Density Lipoprotein Receptor-1 (LOX-1), Proprotein Convertase Subtilisin/Kexin type-9 serine protease (PCSK9), and other specific components, like fatty acid synthase (FASN) [229,230]. These specific components of the lipogenic machinery and cholesterol homeostasis are subject to the regulation of master transcriptional regulators, which may comprise sterol regulatory element-binding proteins (SREBPs) and liver X receptors (LXRs) [231].

### 7.1. Oxidized LDLs and LOX-1 Receptor

#### 7.1.1. Oxidized LDLs

The oxidation of subendothelial LDL, oxLDL, is reported to be a major player in atherosclerosis development [232]. Also, oxLDL has been implicated in many aspects of cancer [229]. OxLDL upregulates HIF-1a expression and increases microRNA miR-210 expression, which leads to the downregulation of sprout-related EVH1 domain 2 (SPRED2), a protein that reduces cell migration, leading to a higher risk of vascular diseases [58]. Downregulation of SPRED2 has been detected in advanced human cancers and is associated with highly metastatic phenotypes [233,234]. OxLDL may disrupt the barrier integrity of the endothelium and represents one of the strongest triggering factors for the transition of endothelial cells into mesenchymal-like cells under pathological conditions, like in the context of atherosclerosis and cancer [235]. The induction of autophagy is an important additional mechanism by which oxLDL participates in cancer progression and promotes EMT when spreading outside the tumor mass [236]. This may be through oxLDL activation of the key metabolic enzyme proline oxidase (POX), which produces superoxide, which exerts its effect by regulating beclin-1 [237].

#### 7.1.2. LOX-1 Receptor

The majority of the atherogenic effects of oxLDL on endothelial function are regulated through the expression and activation of Lectin-Like Oxidized Low-Density Lipoprotein Receptor-1 (LOX-1) [58]. LOX-1 belongs to class E scavenger receptors (SRs) and has the ability to bind to dysfunctional lipids, like oxLDL, fundamental in atherosclerosis and other diseases, like obesity, hypertension, and cancer [238]. LOX-1 is upregulated by many inflammatory mediators and proatherogenic stimuli but has no known enzymatic or catalytic activity, and ligand binding has been shown to trigger intracellular signaling [18,239]. To date, evidence suggests that LOX-1 is involved in a plethora of processes relevant to the pathogenesis of certain malignancies and may play a causative role in tumor initiation and progression [58]. LOX-1 overexpression mediates VEGF induction and HIF-1a activation, promoting neoangiogenic and EMT processes in glioblastoma, osteosarcoma prostate, colon, breast, lung, and pancreatic tumors [240]. In endothelial cells, the binding of oxLDL to LOX-1 increases ROS formation, the PI3K/Akt cascade, and NFkB activation [240,241].

### 7.2. PCSK9 Pathway

#### 7.2.1. Role of PCSK9 in Atherosclerosis

Proprotein convertase subtilisin/kexin type-9 (PCSK9), a serine protease, is now identified as an important and major player in the pathophysiology of atherosclerosis and promotes the onset and progression of CVD [242]. PCSK9 is activated when intracellular cholesterol is reduced and binds to LDL-Rs, redirecting them to lysosomes for cleavage instead of recycling them back to the cell surface, promoting a subsequent increase in LDL-cholesterol (LDL-C) levels [243]. Beyond cholesterol metabolism, other physiological processes are also regulated by PCSK9, such as adipogenesis modulation, the immune response, and interaction with many other cellular receptors, including LOX-1 [244].

PCSK9 can mediate oxLDL-induced inflammation by enhancing the expression of LOX-1, which can increase the uptake of oxLDLs that induce inflammation via activation of NF-kB. PCSK9 can also increase the expression of TLR4, which activates NF-kB to upregulate the expression of inflammatory cytokines, like interleukin 6 (IL-6) [242]. Silencing PCSK9 reduced the expression of inflammatory genes by blocking the TLR4/NFkB pathway in macrophages [245]. PCSK9 increases oxLDL uptake and activates ROS [246]. Given these observations, PCSK9 is clearly involved in the inflammatory response of atherosclerosis.

#### 7.2.2. Role of PCSK9 in Cancer

The recent literature illustrates that PCSK9 is highly expressed and strongly associated with the incidence and progression of most cancers [247]. Τhe use of PCSK9 small interfering RNA (PCSK9i) also enhanced the efficacy of immune therapy targeted at the checkpoint protein PD-1 [248]. Additionally, PCSK9 inhibition has demonstrated the potential to induce cancer cell apoptosis through several pathways, increase the efficacy of a class of existing anticancer therapies, and boost the host immune response to cancer [249]. Hence, a novel application of PCSK9 inhibitors in cancer and metastasis could be considered. However, due to poor data on the effectiveness and safety of PCSK9 inhibitors in cancer, the impact of PCSK9 inhibition on these pathological conditions is still unknown [250].

### 7.3. Sterol Regulatory Element-Binding Proteins (SREBPs)

Sterol regulatory element-binding proteins (SREBPs) are transcription factors for cholesterol production and absorption, and they regulate one of the critical transcription pathways involved in cholesterol homeostasis [251]. Generally, SREBP-1 activates the synthesis of fatty acids and triglycerides, while SREBP-2 increases the synthesis of cholesterol [252]. Activation of SREBP transcription leads to the increased expression of microRNA-33, specifically miR-33a and miR-33b, which are located within intron 16 of SREBP-2 and intron 17 of SREBP-1, respectively [253]. The co-expression of the two miR-33 forms, along with their host genes, can function in a synergistic manner to further facilitate lipid homeostasis [254]. miRNAs are small non-coding RNAs that are key regulators of metabolism and play an important role in cancer by actually downregulating SREBPs in cancer cells [255,256].

The activation of SREBP-1 and -2 ultimately upregulates the expression of enzymes in lipogenesis pathways and the expression of LDLRs, promoting fatty acid and cholesterol synthesis, while LDLR upregulation increases cholesterol uptake [257]. SREBP-1 and -2 also regulate the expression of PCSK9 [258]. SREBP2 was identified as a potent activator of the NLRP3 inflammasome in ECs [259]. In support of the above finding, SREBPs may exacerbate the initiation and progression of atherosclerosis [260].

#### SREBPs in Human Cancers

SREBPs are significantly upregulated in human cancers and mediate a mechanistic link between lipid metabolism reprogramming and malignancy [261]. SREBPs are activated in a lipid-independent manner in cancer by the PI3K/Akt/mTOR/SREBP1 signaling pathway, which is often abnormally activated in tumor cells. Activation of the PI3K/Akt/mTOR signaling pathway induces the transcription of SREBPs, which subsequently promotes cholesterol uptake and synthesis to meet the demand of cancer cells [58]. Moreover, PI3K/Akt/mTOR/SREBP1 signaling protects cancer cells by inhibiting ferroptosis, an iron-dependent form of cell death caused by the accumulation of phospholipid peroxides [262]. SREBP1 inhibits ferroptosis in cancer cells by upregulating its transcriptional target SCD1 and producing monounsaturated fatty acids [263]. In situations in which lipids and/or oxygen is limited, SREBP2 and its downstream targets, including mevalonate-pathway enzymes, are significantly upregulated [264]. The upstream mevalonate pathway is oncogenic in a variety of cancers, mainly in brain tumors like glioblastoma, and requires the oncogene *MYC* for its upregulation [265,266]. This upregulation of the mevalonate pathway further upregulates the microRNA miR-33b; however, it is still not entirely clear whether the lipid accumulation induced by microRNAs through SREBPs has a direct link to the cancer cell phenotype [263]. Whereas oncogene activity promotes cholesterol upregulation, tumor suppressors have the opposite effect. For example, the well-known tumor suppressor *p53* upregulates ABCA1, thereby restricting SREBP2 maturation and repressing the mevalonate pathway [267].

### 7.4. Other Lipogenic Factors in Atherosclerosis and Cancer

Specific components of the lipogenic machinery have been shown to play a pro-tumorigenic role in several cancers. These master transcriptional regulators governing cholesterol homeostasis and lipid metabolism are indispensable for tumor progression and include those discussed below.

#### 7.4.1. Fatty Acid Synthase (FASN)

Fatty acid synthase (FASN) or fatty acid translocase or cluster of differentiation 36 (CD36) is a transmembrane glycoprotein that acts as a downstream molecular complex that facilitates intercellular cholesterol and free fatty acid (FFA) transport [268]. FASN is one of the key components that link lipid synthesis, oxidative stress, and ROS formation, as shown in *p53*-deficient colorectal cancer cells, where ROS-mediated FASN stabilization promotes lipid synthesis and tumor growth [269]. FASN also acts as a signal-transducing receptor for oxLDL [270]. Once FFAs are taken up into the cell, they can be stored in lipid droplets and used for fatty acid β-oxidation and energy production [270]. This may be regulated through the liver kinase B1/LKB1-AMPK pathway [268]. In addition to the exogenous uptake and release of FFAs, FASN could induce insulin resistance and β-cell dysfunction [271]. FASN, as a key downstream target of SREBP, brings fatty acids into cancer cells and is upregulated in breast, prostate, ovarian, stomach, and colorectal cancers and also in many other types of cancers [272].

#### 7.4.2. Further FASN Role in Cancer

High CD36/FASN expression is associated with a poor prognosis in cancers, such as breast, ovarian, gastric, and prostate cancer [85]. Increased FASN expression correlates with established oncogenic events, like human epidermal growth factor receptor 1,2 (HER1/2) amplification in breast cancer, which in turn induces the expression of FASN via activation of PI3K/Akt pathways, resulting in positive feedback to maintain elevated levels of FASN in cancer cells [273]. FASN upregulation and overexpression in cancer cells lead to increased lipid synthesis, in addition to mTOR activation, resulting in increased protein synthesis. FASN also favors the activity of the PPP (pentose phosphate pathway) enzyme PGDH by increasing the pool of NADP+, a co-substrate of the latter. PPP upregulation concomitantly increases DNA/RNA synthesis [274]. The uptake of FFAs (such as oleate and palmitate) via FASN activates oncogenic signaling pathways in liver cancer cells, thereby promoting EMT [275]. FASN/CD36 can also activate the Wnt signaling pathway to promote metastasis through EMT [268].

#### 7.4.3. Liver X Receptors/LXRs

Liver X receptors (LXRs) are transcription factors that belong to the nuclear hormone receptor family. They play a role in lipid metabolism regulation. They act as cholesterol and glucose sensors, ultimately promoting the loss of cellular cholesterol and regulating insulin sensitivity and whole-body homeostasis [276]. LXRs counterbalance the activity of SREBPs, which enhances lipid uptake and biogenesis to maintain cholesterol homeostasis, reversing cholesterol transport and limiting lipid uptake when cellular lipid stores are high [276]. LXRs are nuclear receptors that also modulate intracellular cholesterol levels by upregulating the transcription of efflux proteins such as ATP-binding cassette subfamily A member 1 (ABCA1) and ATP-binding cassette subfamily G member 1 (ABCG1), which are cell membrane proteins that allow cholesterol efflux from cells. ABCA1 is required for high-density lipoprotein (HDL) biogenesis, efflux of cholesterol from macrophages, and reverse transport of cholesterol to the liver [276]. Thus, LXR activity within lesions is atheroprotective [277,278]. Recent studies have demonstrated that TIGAR, which protects against glycolysis and oxidative stress, is associated with ASCVD by upregulating ABCA1 and ABCG1, also interfering with LXR expression via ROS [279]. The activation of LXRs also results in an increase in HIF-1a transcriptional activity [220].

In cancer, LXRs contribute to the development of glioblastoma, a highly lethal brain cancer, which significantly depends on cholesterol and is overly sensitive to LXR-agonist-induced cell death [280]. Also, upregulation of ABCA1 and ABCG1 by LXR agonists has induced apoptosis in prostate and breast cancer cell lines [281]. In tumor immunotherapy, LXR activation therapy produces a strong anti-tumor response in mice and enhances the activation of T cells in various immunotherapy studies, suggesting the LXR/ApoE axis as a target for improving the efficacy of tumor immunotherapy [282]. LXRs contribute to the development of colorectal cancer, along with the enzyme Stearoyl-CoA Desaturase 1 (SCD1), which is directly regulated by LXRs [283]. TIGAR inhibition repressed SCD1 expression in a redox- and AMPK-dependent manner, and TIGAR also induces ferroptosis resistance in colorectal cancer cells via the ROS/AMPK/SCD1 signaling pathway [284].

#### 7.4.4. Stearoyl-CoA Desaturase (SCD)

Desaturation is indispensable in cancer to avoid lipotoxicity under conditions of nutrient stress [272]. Desaturases are controlled by SREBP [272] and by LXRs [283]. In particular, the Stearoyl-CoA desaturase-1 (SCD1) enzyme is a central regulator of lipid metabolism and fat storage. SCD1 catalyzes the generation of monounsaturated fatty acids (MUFAs), which are major components of triglycerides stored in lipid droplets, to form new saturated fatty acid (SFA) substrates—making it a key enzyme involved in finely tuning the MUFA-to-SFA ratio [285].

SCD1 plays an important role in cancer, promoting cell proliferation and metastasis [286]. Its inhibition reduces the MUFA/SFA ratio and contributes to the induction of ferroptosis in tumor cells [287]. Intriguingly, despite LXR agonists eliciting great interest as a promising therapeutic target for cancer, LXR’s ability to induce SCD1 and new fatty acid synthesis represents a major obstacle in the development of new effective treatments [283].

#### 7.4.5. Acetyl-CoA Synthetase 2 (ACSS2)

The acetyl-CoA synthetase (ACSS) enzyme is the sole known mammalian enzyme that can catalyze the conversion of free acetate into acetyl coenzyme A (acetyl-CoA) for lipid synthesis. When cellular cholesterol levels fall below the threshold, proteases begin to act on SREBPs and promote the expression of genes related to cholesterol and fatty acid synthesis, like ACSS2 [288]. SREBP cooperates with the transcription factor LXR, accentuating the importance of ACSS2 in lipid synthesis [288].

More recently, ACSS2 was identified to facilitate the adaptation of cancer cells in the TME by promoting the acetylation of histones and transcription factors and therefore influencing metabolic reprogramming and cell cycle progression in tumors [34,289]. Of interest, to upregulate histone acetylation, ACSS2 forms a complex with TFEB [290]. In addition, studies on tumors have shown that, in cancer cells, ACSS2 interacts with oncoprotein interferon regulatory factor 4 (IRF4) and enhances IRF4 stability and IRF4-mediated gene transcription through the activation of acetylation [291]. Moreover, ACSS2 expression inversely correlates with overall survival in patients with triple-negative breast cancer, liver cancer, glioma, or lung cancer [34].

Below is a summary table of the potential metabolic and oncogenic mechanisms and how they may be involved in cancer initiation and progression (Table 1).

## 8. The Role of Adiposity in Cancer

Human adipose tissue plays functional roles related to triglyceride storage, as well complementary physiological roles in the endocrine system [292]. Adipocyte tissue and its microenvironment may play a role in carcinogenesis, the development of metastases, and the progression of the disease [293]. However, the underlying mechanism resulting in carcinogenesis is complex and not yet fully understood. The main components of the adipose organ include both white adipose tissue (WAT) and brown adipose tissue (BAT) [292,294].

### 8.1. White Adipose Tissue (WAT)

White adipose tissue (WAT) serves many physiological functions, including the storage of lipids for fatty acid supply in a state of energy deprivation, and is involved in a wide array of biological processes that modulate whole-body metabolism and insulin resistance [292]. It stores food calories, creates a layer of thermal insulation, and provides mechanical protection, which is important for resisting infection and injury [295]. Distributed throughout the body, WAT is found near various invasive solid cancers in humans, such as breast, prostate, colon, and kidney cancers and melanoma, and serves as a tremendous reservoir of lipids for cancer cells [294]. However, while the effect of WAT on cancer progression is established and a direct carcinogenic role for WAT cannot be ruled out, there is still a debate about whether WAT actually promotes cancer initiation and, if it does, what mechanisms are involved. A paradigm is that the inflammatory WAT milieu creates an environment in which ROS production is elevated to a level at which genomic instability ensues; however, the role of WAT-generated ROS in tumor initiation remains hypothetical [296]. An antioxidant capacity level that does not cause cell death (redox homeostasis) and mitochondrial function in WAT may be improved by chronic exercise [297]. At this point, there is a clear need to better understand the changes in WAT and the resulting changes in other organs, which underlie cancer progression in obesity, and to understand the role of the conversion of WAT into beige/brown adipose tissue (BAT) in the cancer process [298].

### 8.2. Brown Adipose Tissue (BAT)

Conversely, BAT is a tissue designed for maintaining body temperature at significantly higher levels than ambient temperatures through heat production, primarily via non-shivering thermogenesis [294]. Mediated by the expression of tissue-specific uncoupling protein 1 (UCP1) within the abundant mitochondria, which contributes to its brown appearance, BAT functions to facilitate adaptive thermogenesis, with the uncoupling of ATP production and substrate oxidation [299]. BAT is located in the deep neck region, along large blood vessels, and in the supraclavicular area, and it combusts through β-oxidation of triglyceride-derived fatty acids and glucose, consuming them to produce heat [294]. Naturally, the most potent activator of BAT is cold exposure, which increases sympathetic outflow toward beta-adrenergic receptors in BAT, as was shown by novel experimental evidence from mice, where the exposure of tumor-bearing mice to cold conditions markedly inhibited the growth of various types of solid tumors, including clinically untreatable cancers, such as pancreatic cancers [300]. Research into the bi-directional interactions between adipocytes and cancer cells suggests that adipocytes supply cancer cells with fatty acids for energy production, regardless of which adipose tissue depot they reside in, and cancer cells adapt to the adipose tissue microenvironment by upregulating lipid utilization machinery. However, the association of BAT activation with cancer progression that is evident in rodent models has not yet been tested for clinical relevance. Moreover, it does appear that adipocytes from obese individuals have a more robust tumor-promoting role [38]

### 8.3. Dysfunctional Adiposity and Cancer: The Role of LD Accumulation

Lipid droplets (LDs), also known as lipid bodies or liposomes, are cellular organelles originating from the endoplasmic reticulum, which supplies them with most of their constituent molecules and has a leading role in their biogenesis [301]. They are ubiquitous in cells but are constitutively expressed in fat-storing adipocytes, where they accumulate neutral lipids, including triacylglycerol (TAG) and cholesterol esters (CEs), including fatty acids [302]. LDs are coated with peripheral and integral proteins classified as members of the perilipin (PLIN)-ADRP-TIP47 family or the cell death-inducing DFF45-like effector (CIDE) family lipid metabolism enzymes involved in maintaining lipid homeostasis, such as diacylglycerol acyltransferases 1 and 2 (DGAT1 and DGAT2) [303]. Tyrosine kinase (TKR) ephrin receptor (EPHB2) directly regulates these key proteins involved in maintaining lipid homeostasis [304].

In cancer, LDs appear as metabolic determinants, and their accumulation is now recognized as a key feature of cancer cells. They function in multiple ways to promote cancer progression; however, the mechanisms controlling LD accumulation in cancer are mostly unknown. In particular, LDs release fatty acids to generate acyl-CoA and channel them to mitochondria to produce energy through fatty acid oxidation to boost cancer cell proliferation and metastasis. Moreover, acetyl-CoA can produce NADPH, which acts as a hydrogen donor to maintain redox homeostasis and prevent cell death induced by excessive ROS accumulation [305]. LDs play a vital role in ameliorating endoplasmic reticulum (ER) stress caused by abundant newly synthesized unfolded proteins (UPR) in cancer cells, and the mitochondrial UPR regulates many genes involved in protein folding, ROS defenses, metabolism, the assembly of iron–sulfur clusters, and the modulation of the innate immune response [301,302,306]. In this way, cancer cells employ lipid droplets to ensure redox balance, to initiate autophagy, and to recycle materials from destroyed organelles under metabolic stress, thereby minimizing stress and preventing apoptosis and ferroptosis caused by lipotoxicity and fostering tumor progression. As regulators of (poly)unsaturated fatty acid trafficking, lipid droplets are also emerging as modulators of lipid peroxidation and sensitivity to ferroptosis [307]. Interestingly, tumorigenicity, invasion, and metastasis, as well as chemoresistance, are controlled by a subpopulation of aggressive tumoral cells named cancer stem cells (CSCs), suggesting that LDs may be fundamental elements for stemness in cancer [308]. CSCs are highly tumorigenic and possess a self-renewal capacity and tumor-initiating properties. Among cancers in which lipid molecules are important for CSC tumorigenicity, glioblastoma is recognized as a malignant brain tumor with abundant LDs [308].

## 9. Hyperinsulinemia and Advanced Glycation End Products in Cancer

### 9.1. Hyperinsulinemia and Cancer at Molecular Level

Hyperinsulinemia is an important etiological factor in the development of metabolic syndrome, type 2 diabetes, cardiovascular disease, cancer, and premature mortality [309]. Chronic hyperinsulinemia in type 2 diabetes mellitus activates insulin/insulin-like growth factor-1 (IGF-1) signaling. The binding of insulin to the insulin receptor (IR) leads to the activation of the tyrosine kinase activity of the IR, resulting in tyrosine phosphorylation of the IR substrate and the subsequent activation of the PI3K/Akt pathway [109]. The PI3K/Akt signaling pathway is followed by downstream forkhead box O 1 (FOXO1) protein phosphorylation and mTOR upregulation [310]. The activation of PI3K/Akt/mTOR signaling is responsible for most metabolic and mitogenic effects of insulin by regulating cancer cell survival, proliferation, invasion, migration, differentiation, angiogenesis, and metastasis [109]. As previously noted, TFEB leads to impaired glucose tolerance via reduced Akt signaling and reduced IR substrate 1 and 2 expression [78]. Furthermore, hyperinsulinemia disturbs the balance of the insulin–GH–IGF axis and shifts the insulin–GH ratio toward insulin and away from GH. This insulin–GH shift promotes energy storage and lipid synthesis and hinders lipid breakdown, resulting in obesity due to higher fat accumulation and lower energy expenditure [68]. Moreover, SUMOylation by SUMO (Small Ubiquitin-like Modifier) has emerged as a new mechanism that affects the pathogenesis of type 1 and 2 diabetes mellitus [311]. The dysregulation of the SUMO system is associated with several diseases, particularly cancer. SUMOylation is widely involved in carcinogenesis, the DNA damage response, cancer cell proliferation, metastasis, and apoptosis [311]. Diabetes is one example where combination therapy between immunotherapy and metabolic intervention can be employed to better realize the potential of anticancer therapy. More and more evidence shows that abnormal metabolism in diabetes not only plays a key role in maintaining cancer signals of tumor occurrence and survival but also has a wider significance in regulating the anti-tumor immune response by releasing metabolites and influencing the expression of immune molecules, for instance, lactic acid and arginine [312].

### 9.2. Advanced Glycation End Products (AGEs) and Cancer

The relationship between insulin resistance and hyperglycemia with aberrant signaling overexpression in cancer could be explained by advanced glycation end products (AGEs). AGEs with their Receptor for AGE (RAGE) are involved in the onset and progression of metabolic syndrome [313] and, most importantly, in the pathogenesis of different cancers [314].

AGEs are accumulated active derivatives of high amounts of sugar compounds that interfere with normal amino acids, proteins, lipids, and nucleic acids, a reaction called glycoxidation, disrupting and altering their functionality and activity, as reported by Vlassara H. et al. [315]. Conditions that accelerate AGE accumulation and maintenance include hyperglycemia in diabetes [316]; a diet high in sugar and fat; the consumption of highly processed foods like “fast foods”; cooking at dry, high temperatures, like grilling and frying, compared to cooking in liquid (boiling) [314,317]; artificial-sugar-sweetened beverages [318,319]; and tobacco smoking [320].

There is ample evidence that the AGE–RAGE axis plays a significant role in cancer development, progression, and metastasis, while the overexpression of RAGE was detected in different cancer tissues, especially in highly progressive and metastatic cancers, in accordance with the accelerated glycolytic rate in most aggressive solid cancers [321]. The involvement of the AGE–RAGE system in cancers may include the overexpression of multiple signaling cascades, the activation of oxidative stress and inflammation by ROS, and the interaction of RAGE with RAGE-ligands like HMGB1 (high-mobility group box 1) [322].

First, the interaction between RAGE ligands and RAGE exerts effects on anti- and pro-apoptotic proteins and triggers proinflammatory intracellular signaling cascades through the upregulation of PI3K/Akt/mTOR, MAPKs (mitogen-activated protein kinases), MMPs (matrix metalloproteinases), VEGFs, and NF-κB while downregulating *p53* during cancer progression [323,324]. Other signaling pathways that are activated include ERK (extracellular signal-regulated kinase) and Wnt/β-catenin pathways [314]. RAGE activation of these pathways plays a key role in controlling tumor cells’ proliferation, angiogenesis, and metastatic invasion through the upregulation of NF-κB, which induces proliferation and protects cancer cells from apoptosis [324].

AGE-RAGE, as an oxidant system, promotes the activation of NADPH oxidase, resulting in the formation of ROS, causing further cellular oxidative damage [325]. Ultimately, the establishment of the AGE-RAGE axis leads to the activation of NF-κB, promoting the expression of TGF-β and IL-6, while inducing oxidation in a positive feedback cycle [326,327,328].

Beyond AGEs, RAGE is activated by other ligands, including different inflammation-related molecules, like HMGB1 [323,324]. HMGB1-RAGE exerts a synergistic effect on cancer progression, and HMGB1 overexpression has been documented in cancer tissues of almost all solid tumors: colon, gastric, lung, breast, ovarian, pancreatic, and prostate [135]. In addition to RAGE, TLR2 and TLR4 also bind to HMGB1, which in turn activates NF-κB and PI3K/Akt signaling pathways, conferring protection to cancer cells from apoptosis and directing them toward survival and proliferation [135,322]. HMGB1-RAGE is also found to promote anaerobic glycolysis in fibroblasts, which is required for their activation by breast cancer cells, leading to breast cancer cell metastasis [329]. The interaction of RAGE with its ligands could provide a very interesting target for pharmacological interventions and novel anti-neoplastic agents directed to block RAGE–ligand interactions at the receptor level [330].

## 10. Role of Metabolic Drugs in Lipid Disorders, Diabetes, and Cancer

Dyslipidemia and diabetes, as components of metabolic syndrome, are associated with an increased risk of several types of cancer. Therefore, the use of hypolipidemic and antihyperglycemic medications to lower blood glucose may modify cancer risk. Here, we review available data on the link between the most common classes of hypolipidemic and antihyperglycemic agents and cancer risk among patients with ASCVD and/or metabolic syndrome. The role of drugs in lipid disorders and diabetes to counteract cancer is summarized in Table 2.

### 10.1. Antihyperglycemic Agents and Risk of Cancer

Regarding the association between antihyperglycemic agents and the risk of cancer, recent findings suggest that the risk of cancer associated with the use of antihyperglycemic medications among patients with diabetes depends on the class of drug and type of agent, dosage, and duration of treatment [331]. Among them, the use of insulin and insulin analogs is associated with a significant increase in total cancer risk by almost 50% compared to other antihyperglycemic drugs. Likewise, insulin secretagogues like sulfonylureas have been linked to a ~20% greater risk of cancer, although these associations may be agent-specific and dose-dependent. Alpha-glucosidase inhibitors, e.g., acarbose and thiazolidinediones, have not been consistently associated with cancer [331].

#### 10.1.1. Metformin

Clinical observations support the beneficial effect of metformin on cancer. Metformin is associated with a 20–30% lower risk for all cancers and a greater benefit for cancer-related mortality [331]. It has two main mechanisms to carry out its anti-diabetic and anti-tumorigenic effect: the AMPK-dependent and independent pathways [312]. In the AMPK-dependent pathway, AMPK downregulates insulin/IGF-1, increases glucose uptake, and reduces gluconeogenesis, thus improving glycemic control. Moreover, it inhibits mTORC1, which induces cancer cell proliferation. For the AMPK-independent pathway, metformin regulates oncogenes and tumor suppressor genes, along with anti-tumorigenic effects targeting ROS, NF-kB, and cell cycle regulatory proteins [38,312]. The therapeutic benefit of metformin is underpinned by the potential to reduce the lipotoxicity associated with hypermetabolism and prevent the pathological browning of subcutaneous white adipose tissue [332]. Metformin is also a ligand–RAGE inhibitor [314].

#### 10.1.2. Incretin-Based Therapies

Exposure to distinct types of incretin-based therapies, such as glucagon-like peptide-1 (GLP-1) agonists and dipeptidyl peptidase-4 (DPP-4) inhibitors, has not been found to significantly modify cancer risk [331]. Among GLP-1 agonists, liraglutide exerts cardiovascular protective actions and can preserve endothelial barrier integrity by reversing the oxLDL-induced downregulation of tight junction proteins and attenuating oxLDL-induced endothelial permeability [333]. Furthermore, liraglutide promoted weight reduction, improved angiogenesis in adipose tissue, and alleviated the deleterious effects of aberrant, unhealthy adipose tissue remodeling and metabolic disturbance [334]. There are concerns that liraglutide may promote malignant progression in human triple-negative breast cancer through NADPH oxidase 4 (NOX4) and ROS/VEGF signaling pathways after activating the GLP-1 receptor [335]. However, a recent meta-analysis indicated that liraglutide and other GLP-1 agonists are not associated with an increased risk of breast cancer [336]. Previous data raised concerns about DPP-4 inhibitors’ long-term safety. However, later studies did not confirm these findings. A meta-analysis of site-specific cancers associated with DPP-4 inhibitors did not reveal an elevated cancer risk in DPP-4 inhibitor users [337]. The most recent meta-analysis showed a neutral effect of DPP-4 inhibitors on overall cancer risk, irrespective of the molecule studied and cancer site, and their effect may even be beneficial in the case of colorectal cancer, in which DPP-4 inhibitor use was associated with significantly reduced risk [338].

#### 10.1.3. Inhibitors of Sodium Glucose Cotransporter-2

Long-term data from human studies assessing the impact of SGLT-2 inhibitors on cancer are scarce, and the question regarding whether SGLT-2 inhibitors have anticancer potential or whether they are potentially harmful is still unanswered [339]. The potential for protection against cancer formation and progression from in vitro and animal studies was not confirmed by randomized and observational studies or their meta-analyses [339]. Inhibitors of sodium glucose cotransporter-2 may raise the risk of bladder cancer and reduce the risk of gastrointestinal cancer [331,340].

### 10.2. Hypolipidemic Agents and Risk of Cancer

#### 10.2.1. Statins

Statins inhibit β-Hydroxy-β-methylglutaryl-CoA (HMG-CoA) reductase, which catalyzes the rate-limiting step in hepatic cholesterol biosynthesis and inhibits the mevalonate pathway [341]. Apart from cholesterol biosynthesis, the mevalonate pathway is the key regulator of the synthesis of *Kirsten rat sarcoma viral oncogene homolog* (*KRAS*), which is a critical regulator of the cell cycle and the most frequently mutated oncogene [341]. Statin therapy concomitantly reduces intracellular isoprenoid intermediates such as farnesyl pyrophosphate (FPP) and geranylgeranyl pyrophosphate (GGPP) and post-translational modifications of proteins involved in the proinflammatory response [341,342]. Statins inhibit cancer cell growth by inducing apoptosis, and this is mediated through the inhibition of small guanine triphosphate (GTP)-binding proteins, including Rho, Ras, and Rac proteins [342]. Eventually, this leads to the downregulation of proinflammatory cytokine expression, such as IL-1β [343].

The role of statins in inducing cancer cell death through the inhibition of lipoprotein metabolism via the mevalonate pathway is possible, although this remains unclear, and more studies are needed [344]. Furthermore, statins can inhibit the viability and proliferation of cancer cells by blocking various signaling pathways, such as PI3K/Akt, and may improve the efficiency of chemotherapy when used in combination with other chemotherapeutic agents [58,345]. Notably, statins exert antioxidant effects by modulating the NRF2/HO-1 pathway in different organs and diseases. NRF2 and other proteins involved in NRF2/HO-1 signaling have a crucial role in cellular responses to oxidative stress, which is a risk factor for ASCVD. Statins can significantly increase the DNA-binding activity of NRF2 and induce the expression of its target genes, such as HO-1 and glutathione peroxidase (GPx), thus protecting the cells against oxidative stress [344]. Statins with different solubilities show different effects on cancer therapy. Lipophilic or liposoluble statins have better anticancer activities than hydrophilic statins; this may be partly attributed to their better ability to diffuse into tumors. In this regard, it is proposed that hydrophilic statins are not effective in inhibiting extrahepatic HMG-CoA reductase and are thus ineffective in reducing cancer susceptibility [58].

#### 10.2.2. Ezetimibe

Ezetimibe, on the other hand, is a medication that inhibits intestinal sterol absorption by directly targeting Niemann-Pick C1-like 1 (NPC1L1) [346]. NPC1L1 was found to be associated with the development, pathological stage, and prognosis of colorectal cancer [347]. Murine experimental evidence showed that when compared to high-fat/high-cholesterol-fed animals, ezetimibe reduced breast tumor size and proliferation and inhibited angiogenesis, yielding effects comparable to those seen in mice given a less-fatty/low-cholesterol diet. These findings were followed by a decrease in blood cholesterol levels, but not in intra-tumoral cholesterol levels [348].

#### 10.2.3. PCSK9 Inhibitors

Due to poor data on the effectiveness and safety of PCSK9 inhibitors in cancer, the impact of PCSK9 inhibition on these pathological conditions is still unknown. The recent literature illustrates that PCSK9 is associated with the incidence and progression of several cancers. In several studies, PCSK9 siRNA was shown to effectively suppress the proliferation and invasion of tumor cells. Hence, a novel application of PCSK9 inhibitors/silencers in cancer and metastasis could be considered [249].

### 10.3. Specific Drug Targets of Lipid Metabolism and Cancer

Lipid metabolism is indispensable for tumor progression. Metabolic enzymes are attractive therapeutic targets for cancer therapy, but there has been a paucity of new drugs targeting metabolism for numerous reasons. There are challenges and issues related to both efficacy and safety, which, as for any new medicines, must be optimal for patient benefits. For example, targeting FFA metabolism, the FASN inhibitor (FASNi) platensimycin has an excellent anti-diabetic effect and potential in diabetes-associated breast cancer, especially against the HER2+ subtype [331]. However, efforts to efficiently block FASN for cancer treatment have been hampered by unexpected toxicity and metabolic compensation via lipid uptake [272]. Several SCD1 inhibitors, shown to suppress proliferation and induce apoptosis in a number of cancer cell types, have remained at a pre-clinical level due, at least in part, to mechanism-based adverse events [286]. Among RAGE inhibitors, hispidin, a natural plant product and polyphenol compound, may be able to alleviate cancer progression by counteracting the AGE-RAGE-axis-related induction of carcinogenesis [330]. Hispidin leads to the significant attenuation of AGE formation, RAGE expression, and NF-κB pathway activation through antioxidant activities [349]. Studies showed that hispidin could potentially be a synergistic agent to increase the gemcitabine therapeutic index to treat pancreatic cancer [350]. In addition, hispidin may generate ROS and significantly induce apoptosis in colon cancer cells [351]. NRF2 has emerged as a promising therapeutic target in cancer cells, stimulating extensive research aimed at the identification of natural, as well as chemical, NRF2 inhibitors; however, pharmacological targeting of the NRF2 network is still under investigation [352].

Future clinical trials are needed to quantify the effects of inhibitors of ASCVD on cancer progression, and further basic research is necessary to understand more about the underlying functional mechanisms.

## 11. Final Conclusions

Cancer, a difficult-to-treat disease, may be attributed to error signals of lipid metabolism intertwined with oncogenic mechanisms. Recent investigations have suggested that ASCVD itself may lead to an increased risk of cancer development. To explain the association between ASCVD and cancer, the hypothesis is that atherogenic lipids expose vascular endothelial cells to oxidative stress, ROS production, and an aberrant metabolic switch, which in turn may lead to the activation of oncogenic signals and tumor suppressor gene alterations. Eventually, this may contribute to the initiation and progression of cancer, i.e., especially if a key protective mechanism, like autophagy, is destabilized. However, there is a missing link to bridge the gap between these damaging effects, and data on whether the association between ASCVD and cancer is due to shared risk factors or other mechanisms are conflicting. The correlation between ASCVD and cancer from an epidemiological perspective also remains limited, challenging the validity of this association. The importance of modifiable risk factors that are common to both ASCVD and cancer is also underscored in this equation.

More evident is the role of molecules associated with lipid metabolism in metastatic processes in cancer. Aberrant metabolic mediators, which can facilitate epithelial to mesenchymal transition, cancer invasion, and metastatic spreading, may include Wnt signaling via TGF-β; the upregulation of angiogenic factors, VEGF/VEGFR, and angiopoietins induced by HIF-1a; the induction of autophagy by oxLDL and LOX-1 overexpression; the activation of oncogenic signaling by FASN and SCD1; raised ROS levels; and the uncontrolled activation of the NF-κB pathway. Concerning hyperglycemia in metabolic syndrome and diabetes, emerging evidence links both cancer initiation and dissemination with the RAGE-AGE-HMGB1 system, pointing to novel anticancer drug development.

Nevertheless, a molecular basis is needed to determine the mechanisms by which ASCVD may have an explicit association with the initiation of the process of carcinogenesis. It appears that when clinical observations can be explained at the molecular level, they can be translated back to real-life practice. Most importantly, knowledge of lipid metabolism and cancer may allow novel therapeutic strategies to improve the anticancer response by targeting the common metabolic processes in vascular endothelial cells susceptible to atherogenic lipids and in cancer cells.

After reviewing these molecular mechanisms, we identified several molecular particles, like TFEB, which regulates metabolism in endothelial cells and participates in the regulation of autophagy. In consequence, TFEB enhancement may promote tumorigenesis. Moreover, TP53-induced glycolysis and apoptosis regulator/TIGAR and NRF2 play crucial roles against glycolysis, aberrant inflammation, and cellular responses to oxidative stress; therefore, they may mitigate atherosclerosis. Noteworthy, their elevated expression may be essential for cancer initiation by the ensuing cell damage and malignant transformation. This uncovers TFEB, TIGAR, and NRF2 as potential therapeutic targets for treating various vascular and metabolic diseases, along with cancer. Also, white-to-brown adipose tissue turnover and dysregulated lipid droplets in cancer cells could provide potential therapeutic opportunities in the future.

From this perspective, we are possibly at the beginning of a new era of novel discoveries for sustaining human health.

## Figures and Tables

**Table 1:** Summary table of the potential metabolic driver mechanisms and how they may be involved in cancer.

Relevant Events		In Atherosclerosis	In Cancer
Oncogenic signals	PI3K–Akt–mTOR	Regulates key metabolic processes Regulates glycolysis, OXPHOS, autophagy Is activated in response to insulin to protect against mitogenic effects	Mediates the high demand for cellular nutrients in cancer cells Reprograms cellular metabolism and promotes glycolysis Increases autophagy through enhanced TFEB
	AMPK	Inhibits ROS and foam cell formation LKB1-AMPK activation inhibits fatty acid and cholesterol synthesis Improves insulin sensitivity	Either represses or promotes tumor growth depending on the context
	TLRs	TLR2, TLR4, and TLR9 are involved in endothelial dysfunction and atherosclerosis progression via the expression of inflammatory cytokines	Uncontrolled activation of TLR by chronic inflammatory stimulation with oxLDL, which may ultimately lead to the development of cancer HMGB1, a key ligand for TLRs, provokes inflammatory responses
	NLRP3 inflammasome	OxLDL activates NLRP3, recruits caspase, leading to formation of proatherogenic cytokines (IL-1β) and atheromatous plaque	Warrants further investigations of complicated and contradictory involvement in tumorigenesis Protective role in certain cancers has been shown TRPM2, a regulator of NLRP3, is overexpressed in many cancers
	Notch	DLL4-Notch1 controls the differentiation of macrophages into proinflammatory M1 type involved in the development of atherosclerosis	Associated with different types of cancer Can act as oncogene or tumor suppressor DLL4-Notch implicated in cell-to-cell signaling and angiogenesis in cancer
	Wnt	Controls lipid homeostasis and storage Aberrant Wnt signaling may be important in the pathogenesis of atherosclerosis	Aberrant activation is critical for primary transformation/metastasis Promotes EMT via crosstalk with TGF-β Activates PI3K/Akt, which stimulates HIF-1a-induced metabolic reprogramming
Angiogenic factors	VEGF/VEGFR	Complex and diverse effects VEGF-A may prevent the repair of endothelial damage, contributing to atherogenesis Dysregulation of VEGF-A/VEGFR-1-NRP1 signaling could inhibit chylomicron absorption Decreases the activity of LPL, resulting in accumulation of atherogenic lipoproteins VEGF-B has lipid-lowering effect and, via VEGFR-1/AMPK and NRP-1, controls the uptake of fatty acids by ECs VEGF-B impairs recycling of LDLRs, leading to reduced cholesterol uptake and decrease in GLUT1-dependent endothelial glucose uptake	VEGF/VEGFRs are upregulated in solid tumors, and they significantly contribute to formation of tumor blood vessels, leading to cancer development and dissemination
	Angiopoietins	Ang-1 may be proatherogenic Ang-2, an antagonist of ang-1, may inhibit atherosclerosis by limiting LDL oxidation	Ang-2 is extensively expressed in tumor endothelial cells and triggers tumor angiogenesis Ang-2 augments migration, invasion, and EMT in lung cancer
	NRF2	Considered an important defense mechanism against ASCVD Underlying mechanisms are barely known	Paradoxical roles in cancer, either acting as tumor suppressor or exerting oncogenic effects NRF2 regulates antioxidant response by eliminating ROS Maintains a normal redox state in cancer NRF2 activation, along with TIGAR, supports toxic ROS scavenging NRF2/KEAP1 may also protect against aberrant inflammation, which can result in cell damage and lead to malignant cell transformation
	HIF-1a	Exerts both detrimental and beneficial actions, depending on the cell type expressing HIF May contribute to endothelial cell dysfunction OxLDL induces HIF-1a expression HIF-1a expression depends on ROS	Key regulator in cancer metabolism, induces the switch from OXPHOS to permanent aerobic glycolysis Upregulates angiogenic factors
Lipogenic factors	OxLDL/LOX-1	OxLDL uptake by macrophages leads to foam cell formation and initiation of atherosclerotic plaques Binding of oxLDL to LOX-1 increases ROS LOX-1 mediates oxLDL-induced inflammation	May disrupt endothelial barrier Upregulates HIF-1a and miR-210 Downregulates SPRED2 associated with metastatic phenotypes Trigger factor for EMT Induces autophagy
	PCSK9	Regulates cholesterol metabolism Increases LDL Regulates adipogenesis, immune responses Interacts with LOX-1 and other receptors Increases TLR4 expression	PCSK9 is highly expressed and closely associated with incidence and progression of the majority of cancers
	SREBPs	May exacerbate the initiation and progression of atherosclerosis SREBP-1 activates the synthesis of fatty acids; SREBP-2 increases the synthesis of cholesterol SREBP2 activates NLRP3 in ECs SREBP regulates the expression of PCSK9 and increases miR-33a and miR-33b expression to facilitate lipid homeostasis	SREBPs are significantly upregulated in human cancers They mediate a mechanistic link between lipid metabolism reprogramming and malignancy PI3K/Akt/mTOR/SREBP1 promotes cholesterol uptake in cancer cells PI3K/Akt/mTOR/SREBP1 protects cancer cells from ferroptosis Not entirely clear whether lipid accumulation induced by microRNAs through SREBPs has a direct link to cancer cell phenotype SREBP2 upregulates mevalonate pathway, which is oncogenic
	FASN	Exogenous uptake and release of FFAs Acts as an oxLDL signal-transducing receptor Could induce insulin resistance and β-cell dysfunction	Associated with poor prognosis ROS-mediated FASN promotes lipid synthesis and tumor growth Activates oncogenic signaling like Wnt, promoting EMT and metastasis PI3K/Akt activation, via positive feedback, maintains high levels of FASN in cancer cells
	LXRs	Atheroprotective, promotes HDL biogenesis Upregulates ABCA1 TIGAR interferes with LXR expression Is associated with ASCVD	LXR activation may produce a strong anti-tumor response in mice May contribute to the development of colorectal cancer
	SCD1	Central regulator of lipid metabolism and fat storage Directly regulated by SREBPs and LXRs Catalyzes the generation of MUFAs to form new SFAs	Important role in promoting cancer cell proliferation and metastasis Inhibition reduces MUFA/SFA ratio Induces ferroptosis TIGAR induces ferroptosis resistance in colorectal cancer cells via ROS/AMPK/SCD1 signaling
	ACSS2	Important in lipid synthesis forms a complex with TFEB	Inversely correlates with overall survival in breast cancer Facilitates the adaptation of cancer cells in TME
Adipogenic factors	LDs	Involved in maintaining lipid homeostasis	Recognized as a key feature of cancer Release fatty acids to generate acyl-CoA In mitochondria, through fatty acid oxidation, produce energy to boost cancer cell proliferation and metastasis Synthesis of UPR in mitochondria regulates ROS defenses and metabolism and ensures redox balance Highly aggressive CSCs are abundant in LDs of some cancer types

Note: PI3K-Akt, phosphoinositide 3-kinase—protein kinase B; TFEB, endothelial transcription factor EB; OXPHOS, oxidative phosphorylation; AMPK, adenosine monophosphate-activated protein kinase; TLRs, toll-like receptors; NLRP3, Nod-like receptor protein 3 inflammasome; HMGB1, high-mobility group box 1; TRPM2, transient receptor potential melastatin 2; TGF-β, transforming growth factor-β; VEGF/VEGFRs, endothelial growth factor/VEGF receptors; NRF2, nuclear factor erythroid 2-related factor 2; HIF-1a, hypoxia-inducible factor-1a; oxLDL, oxidized LDL; LOX-1, Lectin-Like Oxidized Low-Density Lipoprotein Receptor-1; SREBPs, sterol regulatory element-binding proteins; FASN, fatty acid synthase; LXRs, liver X receptors; SCD, stearoyl-CoA desaturase; ACSS2, acetyl-CoA synthetase 2; LPL, plasma lipoprotein lipase; ECs, endothelial cells; LDLR, low-density lipoprotein receptor; NRP1, Neuropilin 1; GLUT1, glucose transporter 1; EMT, epithelial–mesenchymal transition; TIGAR, TP53-induced glycolysis and apoptosis regulator; KEAP1, Kelch-like ECH-associated protein 1; SPRED2, sprout-related EVH1 domain 2; LOX-1, Lectin-Like Oxidized Low-Density Lipoprotein Receptor-1; SREBPs, Sterol regulatory element-binding proteins; miR, microRNA; FFAs, free fatty acids; HDL, high-density lipoprotein; ABCA1, ATP-binding cassette subfamily A member 1; MUFAs, monounsaturated fatty acids; SFA, saturated fatty acid; TME, tumor microenvironment; LDs, lipid droplets; acyl-CoA, acyl-CoA synthetase; UPR, unfolded proteins; CSCs, cancer stem cells.

**Table 2:** Role of drugs in lipid disorders and diabetes to counteract cancer.

Drug Category	Type of Drug	Mechanisms	Role in Cancer
Antihyperglycemic agents	Metformin	Downregulates insulin/IGF-1 through AMPK Inhibits cancer cell proliferation by mTORC1 inhibition Regulates oncogenes and tumor suppressor genes Targets ROS Prevents lipotoxicity and the pathological browning of WAT Ligand RAGE inhibitor	Beneficial effect
	GLP-1 agonists, liraglutide as example	Cardiovascular protective actions Could preserve endothelial barrier integrity by reversing oxLDL-induced endothelial permeability	Has not been found to significantly modify cancer risk
	DPP-4		Neutral effect on overall cancer risk; may even be beneficial in colorectal cancer—significantly reduced risk
	SGLT-2		Unclear whether it possesses anticancer potential or if it is potentially harmful May raise risk of bladder cancer and reduce risk of gastrointestinal cancer
	Insulin and insulin analogs		Associated with a significant increase in total cancer risk by almost 50% compared to other antihyperglycemic drugs
Hypolipidemic agents	Statins	Inhibit HMG-CoA reductase, involved in cholesterol biosynthesis, and inhibit the mevalonate pathway Antioxidant effects by modulating NRF2/HO-1 Block the proliferation of cancer cells by inhibiting PI3K-Akt Inhibit cancer cell growth by inducing apoptosis mediated through inhibition of GTP Inhibit the mevalonate pathway Possibly induce cancer cell death, although this still remains unclear	May inhibit cancer cell growth Lipophilic statins have better anticancer activities
	Ezetimibe	Inhibits intestinal sterol absorption by directly targeting NPC1L1	Ezetimibe reduced breast tumor size and proliferation in mice NPC1L1 can serve as an independent prognostic marker for colorectal cancer
	PCSK9 inhibitors	induce cancer cell apoptosis	Poor data on effectiveness and safety of PCSK9 inhibitors in cancer still unknown role PCSK9 siRNA may suppress the proliferation and invasion of several tumors
Specific drug targets of lipid metabolism	FASN inhibitors (FASNi)	Target FFA metabolism FASNi platensimycin has anti-diabetic effect and potential in diabetes-associated breast cancer, especially against the HER2+ subtype	Unexpected adverse events
	SCD1 inhibitors	Suppress proliferation and induce apoptosis in a number of cancer cell types	Have remained at a pre-clinical level
	LXRs agonists		Strong anti-tumor response in mice The development of new effective treatments is hampered because LXRs induce SCD1 and fatty acid synthesis
	NRF2 inhibitors	Maintaining a normal redox state can have a detrimental impact on cancer treatment	Still under investigation

Note: IGF-1, insulin-like growth factor-1; RAGE, Receptor for advanced glycation end products (AGEs); WAT, white adipose tissue; GLP-1, glucagon-like peptide-1 agonists; DPP-4, dipeptidyl peptidase-4 inhibitors; SGLT-2, inhibitors of sodium glucose cotransporter-2; HMG-CoA, β-Hydroxy-β-methylglutaryl-CoA reductase; GTP, small guanine triphosphate-binding proteins; NRF2/HO-1, nuclear factor erythroid 2-related factor 2/heme oxygenase-1; NPC1L1, Niemann-Pick C1-like 1; other abbreviations are mentioned in Table 1.

## Data Availability

This manuscript generated no new datasets.
